# Characterization of purple acid phosphatases involved in extracellular dNTP utilization in *Stylosanthes*

**DOI:** 10.1093/jxb/erw190

**Published:** 2016-05-18

**Authors:** Pan-Dao Liu, Ying-Bin Xue, Zhi-Jian Chen, Guo-Dao Liu, Jiang Tian

**Affiliations:** ^1^College of Agriculture, Hainan University, Institute of Tropical Crop Genetic Resources, Chinese Academy of Tropical Agriculture Sciences, Hainan 570228, P. R. China; ^2^State Key Laboratory for Conservation and Utilization of Subtropical Agro-bioresources, Root Biology Center, South China Agricultural University, Guangdong 510642, P. R. China

**Keywords:** Acid phosphatase activities, dNTP, organic phosphorus, phosphate starvation, purple acid phosphatase, *Stylosanthes*.

## Abstract

Stylo (*Stylosanthes* spp.) is a pasture legume predominant in tropical and subtropical areas, where low phosphorus (P) availability is a major constraint for plant growth. Therefore, stylo might exhibit superior utilization of the P pool on acid soils, particularly organic P. However, little is known about mechanisms of inorganic phosphate (Pi) acquisition employed by stylo. In this study, the utilization of extracellular deoxy-ribonucleotide triphosphate (dNTP) and the underlying physiological and molecular mechanisms were examined for two stylo genotypes with contrasting P efficiency. Results showed that the P-efficient genotype, TPRC2001-1, was superior to the P-inefficient genotype, Fine-stem, when using dNTP as the sole P source. This was reflected by a higher dry weight and total P content for TPRC2001-1 than for Fine-stem, which was correlated with higher root-associated acid phosphatase (APase) activities in TPRC2001-1 under low P conditions. Subsequently, three *PAP* members were cloned from TPRC2001-1: *SgPAP7*, *SgPAP10*, and *SgPAP26*. Expression levels of these three *SgPAPs* were up-regulated by Pi starvation in stylo roots. Furthermore, there was a higher abundance of transcripts of *SgPAP7* and *SgPAP10* in TPRC2001-1 than in Fine-stem. Subcellular localization analysis demonstrated that these three SgPAPs were localized on the plasma membrane. Overexpression of these three *SgPAPs* could result in significantly increased root-associated APase activities, and thus extracellular dNTP utilization in bean hairy roots. Taken together, the results herein suggest that SgPAP7, SgPAP10, and SgPAP26 may differentially contribute to root-associated APase activities, and thus control extracellular dNTP utilization in stylo.

## Highlight

SgPAP7, SgPAP10, and SgPAP26 may differentially contribute to root-associated APase activities, and thus control extracellular dNTP utilization in stylo.

## Introduction

Phosphorus (P) is an essential macronutrient for plant growth and development. Inorganic phosphate (Pi) is the major form of P acquired by plants directly from soil (Marschner, 1995). However, Pi is easily fixed by soil particles and microorganisms, thus turning into unavailable forms (e.g. Fe/Al-P complexes and organic P), which cannot be directly utilized by plants (Lambers and Plaxton, 2015). It has been estimated that 30–65% of total P in soils is present as organic P, mainly in the form of phytate, nucleic acids, and phospholipids (Shen *et al.*, 2011; Stutter *et al.*, 2012). As a result, low Pi availability is a major constraint limiting plant growth and production on both natural and agricultural soils (Vance *et al.*, 2003; López-Arredondo *et al.*, 2014).

Plant adaptations to improve Pi acquisition and utilization under conditions of P deficiency lie within a wide-ranging set of available morphological, physiological, and molecular strategies, such as modification of root morphology and architecture (Lynch, 2015), increased organic acid and acid phosphatase (APase) secretion (Plaxton and Tran, 2011; Shen *et al.*, 2011; Song and Liu, 2015; Tian and Liao, 2015), and enhanced high-affinity Pi transporter expression (Qin *et al.*, 2012; Zhang *et al.*, 2015). It has recently been established that the adaptive strategies listed above are tightly and coordinately mediated through a variety of regulators in complex P signalling networks (Chiou and Lin, 2011; Wu *et al.*, 2013; Liang *et al.*, 2014). Among the adaptive responses, increased APase activities and exudation is generally considered as a means for plants to mobilize and utilize organic P (Tran *et al.*, 2010a; Tian and Liao, 2015).

Plant APases (EC 3.1.3.2) belong to the hydrolase enzymes, which catalyse phosphate from a group of phosphomonoesters and anhydrides with optimal activity pH below 7.0 (Duff *et al.*, 1994). Among the APases, purple acid phosphatase (PAP) has distinctive biochemical and molecular characteristics, including a purple or pink colour in aqueous solution, activity that is insensitive to inhibition by L-tartrate, a bimetallic active site, and the presence of seven invariant residues in five conserved metal ligating motifs (Bozzo *et al.*, 2004; Schenk *et al.*, 2013; Matange *et al.*, 2015; Tian and Liao, 2015). PAPs have been widely identified and characterized from a range of organisms, including mammals, plants, fungi, and bacteria (Schenk *et al*., 2000a, 2000b, 2013; Flanagan *et al.*, 2006). Despite low homology between PAPs from different kingdoms, five conserved motifs have been identified, including ***D***XG, G***D***XX***Y***, G***N***H(D/E), VXX***H***, and G***H***X***H*** (Oddie *et al.*, 2000; Schenk *et al*., 2000a, 2000b, 2013; Flanagan *et al.*, 2006; Mitić *et al.*, 2006). Within the conserved motifs, these seven invariant residues shown in bold and italic have been suggested to participate in the formation of a binuclear centre by coordinating with Fe^3+^ and other divalent metal ions, such as Fe^2+^, Mn^2+^, and Zn^2+^ (Beck *et al.*, 1986, 1988; Sträter *et al.*, 1995; Schenk *et al.*, 1999, 2005; Mitić *et al.*, 2006). Unlike animal PAPs, the second divalent metal ion in non-recombinant plant PAP members varies among plant species. In red kidney bean (*Phaseolus vulgaris*), soybean (*Glycine max*), and sweet potato (*Ipomoea batatas*), PAPs containing a Fe^3+^–Zn^2+^ centre have been identified (Beck *et al.*, 1986; Durmus, *et al.*,1999a; Schenk *et al.*, 1999). Another PAP from sweet potato contains a Fe^3+^–Mn^2+^ centre (Schenk *et al.*, 1999, 2001, 2005). Biochemical analysis of recombinant and non-recombinant PAPs has documented that most plant PAPs can catalyse the hydrolysis of a wide range of phosphorylated compounds both natural and synthetic, such as ρ-nitrophenyl phosphate, energetic compounds (e.g. ATP and inorganic pyrophosphate), phosphorylated sugars, and phosphorylated amino acids (Tran *et al.*, 2010a; Tian and Liao, 2015). However, NtPAP from tobacco (*Nicotiana tabacum*) and AtPAP15 from Arabidopsis (*Arabidopsis thaliana*) exhibit relatively high rates of activities with phytate as the substrate (Lung *et al.*, 2008; Zhang *et al.*, 2008). Among plant pioneer PAP studies, one PAP from kidney bean has been well characterized from its protein structure to catalytic properties (Beck *et al.*, 1986, 1988; Klabunde *et al.*, 1994; Sträter *et al.*, 1995). Subsequently, other plant PAPs have been purified, and their amino acid sequences elucidated in plant species including soybean, sweet potato, and Arabidopsis (Del Pozo *et al.*, 1999; Durmus *et al.*, 1999b; Schenk *et al.*, 1999, 2000a). The conserved sequence patterns of PAPs facilitated the identification of a large number of novel PAP and PAP-like sequences in plants (Schenk *et al.*, 2000a; Li *et al.*, 2002). Furthermore, with the availability of genomic sequences for diverse plant species, PAP members have now been widely identified and characterized in plants such as Arabidopsis, rice (*Oryza sativa*), soybean, and maize (*Zea mays*). Phosphate starvation has been documented to increase *PAP* expression levels for 11 out of 29 members in Arabidopsis, 10 out of 26 in rice, 23 out of 35 in soybean, and 11 out of 33 in maize (Li *et al.*, 2002; Zhang *et al.*, 2011; Li *et al.*, 2012a; Wang *et al.*, 2014; González-Muñoz *et al.*, 2015). These results suggest that many plant *PAP* members are involved in P scavenging and recycling under conditions of P deficiency. This hypothesis is supported by functional analysis results for a number of plant *PAP* members. For example, overexpression of *AtPAP15* from Arabidopsis in soybean, *MtPHY1* from *Medicago truncatula* in white clover (*Trifolium repens*), and *OsPHY1* from rice in tobacco could significantly increase transgenic plant dry weight and total P content when phytate was supplied as the sole P source (Ma *et al.*, 2009; Wang *et al.*, 2009; Li *et al.*, 2012b). In addition, several other *PAP* members appear to participate in the utilization of other forms of extracellular organic P, such as ATP, ADP, and dNTP (Liang *et al.*, 2010, 2012; Wang *et al.*, 2011; Robinson *et al.*, 2012). These results strongly suggest that root-secreted PAP has a pivotal role in the scavenging and recycling of extracellular P. Still, comprehensive dissection of PAP functions remains elusive owing to the variation in gene and protein structures, regulation of expression at multiple levels (i.e. transcriptional and post-translational), and the localization in a wide range of subcellular and extracellular compartments (Tian and Liao, 2015). The ability of plant PAPs to serve in multiple and complex functions is evident in recent findings, such as participation of GmPAP3 in salt stress tolerance in soybean (Liao *et al.*, 2003; Li *et al.*, 2008), NtPAP12 in cell wall biosynthesis in tobacco (Kaida *et al.*, 2009), and AtPAP2 in carbon metabolism and AtPAP5 in biotic stress resistance (Sun *et al.*, 2012; Ravichandran *et al.*, 2013; Law *et al.*, 2015). These results suggest that integrating all available knowledge of PAP properties will be critical for further understanding the complex range of PAP functions related to P scavenging and recycling in plants (Tian and Liao, 2015).

Stylo (*Stylosanthes* spp.) is a dominant pasture legume that is widely grown in tropical and subtropical areas, where acid soils are widely distributed (Chandra, 2009). On acid soils, P deficiency, aluminium (Al) toxicity, and manganese (Mn) toxicity are considered as major constraints for crop growth and production (Kochian *et al.*, 2004). Therefore, it is possible that stylo is capable of overcoming these constraints to grow on acid soils. Recently, it has been documented that malate synthesis and exudation in stylo allow it to tolerate both Al and Mn toxicity (Sun *et al.*, 2014; Chen *et al.*, 2015). Even so, wide variation in P efficiency has been observed among stylo genotypes (Du *et al.*, 2009). To date, physiological and molecular mechanisms underlying stylo adaptation to P deficiency remain largely unknown. In this study, dNTP utilization was investigated between two stylo genotypes with contrasting P efficiency. Subsequently, three *SgPAP* members were cloned and characterized to elucidate molecular mechanisms of dNTP utilization in stylo.

## Materials and methods

### Plant material and growth conditions

Two *Stylosanthes guianensis* Aubl. genotypes with contrasting P efficiency, TPRC2001-1 (P-efficient genotype) and Fine-stem (P-inefficient genotype), were used in this study. Seeds of stylo were soaked in 80°C water for 2min before being surface sterilized in 10% (v/v) sodium hypochlorite solution and sown in solidified Murashige and Skoog (MS) medium lacking KH_2_PO_4_. After germination, seedlings with 1 cm-long roots were transferred to 1.2mM KH_2_PO_4_ (+P), 0.4mM dNTP (dNTP), or no added P (−P) treatments on solid MS medium. For dNTP treatments, the medium was autoclaved and supplemented with 0.4mM filter sterilized dNTP, containing 0.1mM each of dATP, dGTP, dTTP, and dCTP. Plates were placed vertically in growth chambers with a 16/8h (light/dark) photoperiod (100 μmol m^−2^ s^−1^ photosynthetically active radiation) at 23°C. After 7 d growth, shoots and roots were separately harvested to analyse dry weight, soluble Pi concentration, APase activities, and total P content. Soluble Pi concentration and total P content were assessed using the phosphorus-molybdate blue colour reaction method according to Murphy and Riley (1962).

### Cloning and characterization of *SgPAP7*, *SgPAP10*, and *SgPAP26*

To clone homologues of *PvPAP3*, *AtPAP10*, and *AtPAP26* in stylo, primers (*SgPAP7/10/26*-EST-F and *SgPAP7/10/26*-EST-R, Supplementary Table S1) were designed separately according to the conserved motif sequences of *PvPAP3* (NCBI accession FJ464333.1), *AtPAP10* (NCBI accession NM_127196.3), *AtPAP26* (NCBI accession NM_122874.3), and three *GmPAPs* (NCBI accessions NM_001254279.1, NM_001253997.1, NM_001249748.1). Three fragments were successfully amplified by PCR from the cDNA of TPRC2001-1 plants subjected to P deficiency, and then cloned into the *pMD18-T* vector (Takara, Japan). Subsequently, the 5′ and 3′ terminals of the three *PAP* fragments were amplified from a TPRC2001-1 RACE cDNA library constructed with the SMARTer™ RACE cDNA amplification kit (Clontech, USA) using specific primers paired with the universal primers RACE-UPM and RACE-NUP (Supplementary Table S1). After separately cloning 5′-terminal and 3′-terminal cDNA products into the *pMD18-T* vector, full-length sequences of *SgPAP7*, *SgPAP10*, and *SgPAP26* were generated through the MEGA 5 program. The nucleotide sequences of these three *SgPAPs* were deposited in NCBI and assigned the accession numbers KU315544, KU315545, and KU315546 for *SgPAP7*, *SgPAP10*, and *SgPAP26*, respectively. Multiple sequence alignments and phylogenetic trees were constructed in ClusterX and MEGA 5, respectively. Signal peptides were predicted by SignalP 4.1 (http://www.cbs.dtu.dk/services). The transmembrane topology of the proteins was predicted by MEMSATSVM (http://bioinf.cs.ucl.ac.uk/web_servers/).

### APase activity analysis

Acid phosphatase activity assays were performed as described by Liang *et al.* (2012). Briefly, to measure internal APase activities, protein extracted from plant tissues was mixed with 2mL of 45mM Na-acetate buffer (pH 5.0) containing 1mM ρ-nitrophenyl phosphate (ρ-NPP; Sigma, USA). After incubation at 37°C for 15min, the reaction was stopped by adding 1mL of 1M NaOH. APase activities were measured spectrophotometrically as absorbance at 405nm. Soluble protein concentration was analysed using the Coomassie Brilliant Blue method (Bradford, 1976). APase activities are presented as micromoles of ρ-NPP hydrolysed per milligram of soluble protein per minute.

For quantitative analysis of root-associated APase activities, roots of stylo or transgenic bean hairy roots were incubated in 45mM Na-acetate buffer containing 2mM ρ-NPP (pH 5.0). After incubation for 30min, the reaction was terminated by adding 1mL of 1M NaOH, and the absorbance of the reaction mix was measured at 405nm. Root-associated APase activities are expressed as micromoles of ρ-NPP hydrolysed per hour per centimetre of root.

For root-associated APase activity staining, transgenic bean hairy roots were placed on solid MS medium. These roots were then evenly overlaid with 0.5% (w/v) agar solution containing 0.02% (w/v) of the substrate 5-bromo-4-chloro-3-indolyl-phosphate (BCIP; Sigma, USA) for 2h at 25°C. Root-associated APase activities were visualized as the intensity of blue colour along root surfaces after BCIP hydrolysation (Lloyd *et al.*, 2001; Wang *et al.*, 2011). Images were captured using a single lens reflex camera (Canon, Japan). Root-associated APase activities were also investigated using ELF-97 phosphate as a substrate according to previous methods (Robinson *et al.*, 2012). Briefly, transgenic bean hairy roots were rinsed three to five times with 45mM Na-acetate buffer (pH 5.0) and incubated in this buffer with 100 µM ELF-97 phosphate (Invitrogen, USA) for 30min at 25°C. After incubation, transgenic bean hairy roots were rinsed three times with 45mM Na-acetate buffer (pH 5.0) containing 25mM EDTA for 15min. The green fluorescent product of root-associated APase activities was imaged using a fluorescence microscope (Leica, Germany) with excitation at 345nm, and emission at 530nm. Images of root-associated APase activity staining were collected from three independent replicates, and representative results are shown.

### Subcellular localization of SgPAPs

To analyse the subcellular localization of SgPAP7, SgPAP10, and SgPAP26, the open reading frame (ORF) sequences of *SgPAP7*, *SgPAP10*, and *SgPAP26* without stop codons were amplified from cDNA stock of TPRC2001-1 with specific primers (Supplementary Table S1). Subsequently, these three *SgPAP* sequences were separately cloned into the binary vector *pLGFP* and fused with GFP at the C-terminus. The *SgPAP-GFP* fusion vectors and *GFP* empty constructs were separately introduced into *Agrobacterium tumefaciens* strain GV3101. Transformed GV3101 cells were grown overnight at 28°C in liquid yeast extract peptone (YEP) medium, and suspended to an absorbance of 0.5 at 600nm in agroinfiltration buffer (pH 5.6), containing 10mM MES, 10mM MgCl_2_, and 0.15mM acetosyringone. After incubating for 3h at 25°C in the dark, equal volumes of mixed suspensions were syringe-infiltrated into the abaxial side of near-fully expanded leaves of 5–6-week-old tobacco (*Nicotiana benthamiana*) plants. After 3 d, epidermal cells on the abaxial leaf side were imaged on a Zeiss LSM7 DUO confocal microscope (Zeiss, Germany). Co-localization experiments were performed using the mCherry-labelled plasma membrane marker *AtPIP2A*-mCherry.

For subcellular localization of SgPAPs in transgenic bean hairy roots, the *SgPAP-GFP* fusion and *GFP* empty constructs were separately transformed into *Agrobacterium rhizogenes* strain K599, which were then used to generate transgenic bean hairy roots with *SgPAP-GFP* or *GFP* overexpression. Transgenic bean hairy roots were generated as described by Liang *et al.* (2012). Confocal images were taken on a Zeiss LSM7 DUO confocal microscope (Zeiss, Germany). GFP fluorescence was stimulated at 488nm and detected with filter sets at 500–530nm. For western blot analysis, soluble proteins and membrane proteins were separately extracted using the FOCUS™ Global Fractionation kit (G-Biosciences, USA) from transgenic hairy roots, which were generated as above. Western blotting analysis was carried out as described by Liang *et al.* (2010). Briefly, extracted proteins were separated by 10% SDS-PAGE in a Mini-PROTEAN Tetra Cell (Bio-Rad, USA) and transferred onto polyvinylidene difluoride membranes (GE Healthcare Life Sciences, USA) using a Trans-Blot Cell (Bio-Rad, USA). Subsequently, the membranes were incubated overnight in 0.01M Tris buffer (pH 8.0) containing 5% (w/v) non-fat milk powder and 0.15M NaCl. Membranes were separately probed with a GFP antibody (HuaAn Biotechnology, China), a phosphoenolpyruvate carboxylase antibody (Agrisera, Sweden), and a plasma membrane proton ATPase antibody (Agrisera, Sweden). Antigenic polypeptides were visualized using alkaline-phosphatase-tagged secondary antibodies and BCIP/NBT substrates. For localization in Arabidopsis protoplasts, the *SgPAP-GFP* fusion or *GFP* empty constructs were transiently expressed in Arabidopsis mesophyll protoplasts following the methods of Wu *et al.* (2009). The GFP fusion constructs were co-transfected with the plasma membrane marker *AtPIP2A*-mCherry. Confocal images were taken on a Zeiss LSM7 DUO confocal microscope (Zeiss, Germany). GFP fluorescence was stimulated at 488nm and detected with filter sets at 500–530nm. The mCherry fluorescence was excited at 568nm and emission captured at 580–630nm.

### Quantitative real-time PCR analysis

Total RNA was extracted using TRIzol reagent (Invitrogen, USA). DNase I-treated RNA (2 µg) was used for first-strand cDNA synthesis by M-MLV reverse transcriptase (Promega, USA) according to the manufacturer’s manual. The quantitative real-time PCR (qRT-PCR) analysis was carried out using SYBR Premix ExTaq II (Takara, Japan) on the Rotor-Gene 3000 qRT-PCR system (Corbett Research, Australia). The housekeeping genes *SgEF-1a* (accession number, JX164254) and *PvEF-1a* (PvTC3216 from the Dana-Farber Cancer Institute Computational Biology and Functional Genomics Laboratory) were used as internal controls to normalize gene expression for stylo and bean hairy roots, respectively. The primer pairs for amplification of *SgPAPs* and the housekeeping gene used for qRT-PCR analysis are listed in Supplementary Table S1. All of the gene expression analyses had four biological replicates.

### Functional analysis of *SgPAPs* in bean hairy roots

To overexpress *SgPAP7*, *SgPAP10*, and *SgPAP26* in bean hairy roots, the ORFs of *SgPAP7*, *SgPAP10*, and *SgPAP26* were amplified from cDNA stock of TPRC2001-1 using gene-specific primers (see Supplementary Table S1), and then cloned into the *pYLRNAi* binary vector. The constructed vectors were transferred into *Agrobacterium rhizogenes* strain K599, which was used for bean hairy roots transformation and induction as described previously by Liang *et al.* (2012). Transgenic hairy roots were verified by qRT-PCR analysis.

In the dNTP utilization experiments, approximately 0.1g (fresh weight) of hairy roots was cultured in solid MS medium supplied with 0 µM KH_2_PO_4_ (−P), 1.2mM KH_2_PO_4_ (+P), or 0.4mM dNTP (i.e. 0.1mM each of dATP, dGTP, dTTP, and dCTP) as the P sources. After 14 d growth, transgenic bean hairy roots were photographed using a single lens reflex camera (Canon, Japan). Fresh weight and total P content of each transgenic line were determined as described above. Each treatment had four biological replicates.

### Statistical analysis

All the data were analysed by ANOVA or *t*-test using SPSS software (version 18.0, SPSS Institute, USA).

## Results

### Utilization of exogenous dNTP in stylo

**Fig. 1.**
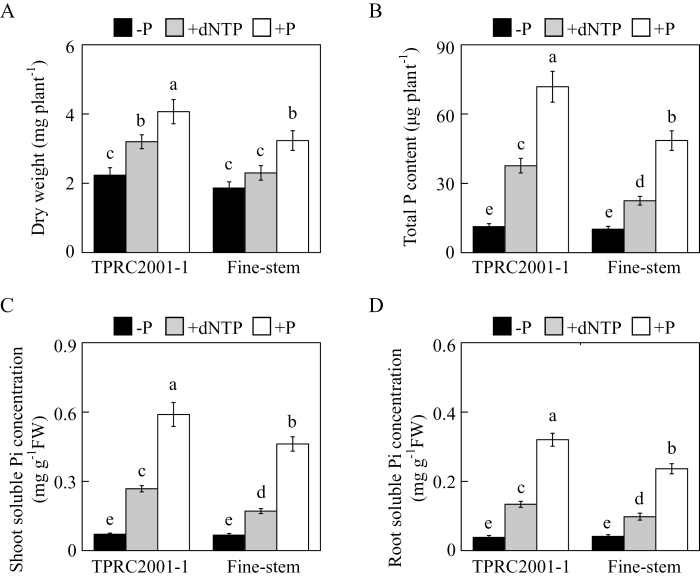
Effects of different P sources on plant growth and Pi acquisition in stylo. (**A**) Dry weight. (**B**) Total P content. (**C**) Soluble Pi concentration in shoots. (**D**) Soluble Pi concentration in roots. After seed germination, seedlings with 1cm root length were grown in MS medium containing 0 µM (−P), 1.2mM KH_2_PO_4_ (+P), or 0.4mM dNTP (+dNTP) as the sole P source. After 7 d, dry weight, total P content, and soluble Pi concentration were separately measured. Each bar shows the mean of four replicates with standard error. Significant differences (*P* < 0.05) are indicated by different letters. FW, fresh weight.

A P-efficient genotype, TPRC2001-1, and a P-inefficient genotype, Fine-stem, of stylo were used to investigate the capacity of genotypes to utilize extracellular dNTP. As expected, plant growth was significantly affected by P source (Fig. 1). Stylo growth was significantly inhibited, with the lowest plant dry weight and total P content found in the −P treatment (Fig. 1A, B). With application of dNTP or phosphate, both plant dry weight and total P content significantly increased, except for dry weight of Fine-stem in dNTP. With dNTP application, plant dry weight and total P content were higher in TPRC2001-1 than in Fine-stem by 39% and 68%, respectively (Fig. 1A, B). Consistent with these results, Pi concentration in both shoots and roots of both stylo genotypes was higher in dNTP than in −P. Again, genotypic variation was observed with dNTP application, with a higher soluble Pi concentration in TPRC2001-1 than Fine-stem for both shoots and roots, by 56% and 36%, respectively (Fig. 1C, D). Taken together, these results strongly suggest that TPRC2001-1 is more capable of utilizing dNTP as a P source than Fine-stem.

### Root internal and root-associated APase activities at two P levels

**Fig. 2.**
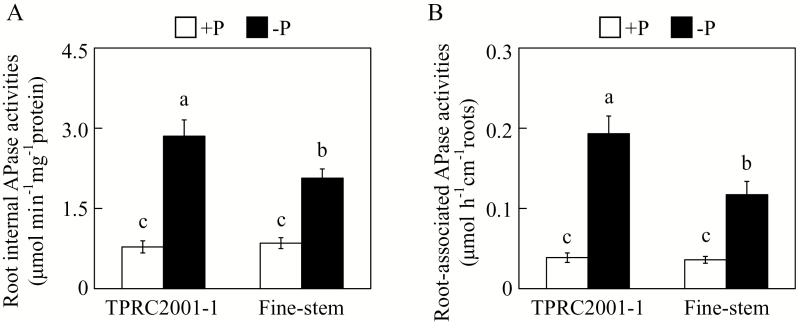
APase activities in roots of two stylo genotypes at two P levels. (**A**) Root internal APase activities. (**B**) Root-associated APase activities. After seed germination, seedlings with 1cm root length were grown in MS medium containing 1.2mM KH_2_PO_4_ (+P) or 0 µM (−P). After 7 d, root internal and root-associated APase activities were separately measured. Each bar represents the mean of four independent replicates with standard error. Significant differences (*P* < 0.05) are indicated by different letters.

To examine whether APase participates in extracellular dNTP utilization, both root internal and root-associated APase activities were assayed in the two stylo genotypes. Both root internal and root-associated APase activities were significantly increased by P deficiency in both stylo genotypes (Fig. 2A, B). Furthermore, root internal and root-associated APase activities in TPRC2001-1 were 38% and 65% higher than those in Fine-stem under low P conditions, despite the fact that no differences in root APase activities were observed between two stylo genotypes under high P conditions (Fig. 2A, B). This strongly suggests that the different capabilities of the two stylo genotypes to utilize extracellular dNTP might be caused by them having different root APase activities.

### Isolation and bioinformatic analysis of three stylo SgPAPs

Because extracellular dNTP utilization has been documented for PvPAP3 in bean, as well as for AtPAP10 and AtPAP26 in Arabidopsis (Liang *et al*., 2010, 2012; Wang *et al.*, 2011, 2014; Robinson *et al.*, 2012), full-length cDNA of homologs of *PvPAP3*, *AtPAP10*, and *AtPAP26* in stylo were cloned and named *SgPAP7*, *SgPAP10*, and *SgPAP26*, respectively. *SgPAP7*, *SgPAP10*, and *SgPAP26* contain 993-bp, 1401-bp, and 1431-bp ORFs, which encode polypeptides with 330, 466, and 476 amino acid residues, respectively (see Supplementary Fig. S1). Bioinformatic analysis predicted that all three SgPAPs have an N-terminal signal peptide (Supplementary Fig. S1). Multiple sequence alignment of SgPAP7, SgPAP10, and SgPAP26 amino acid sequences with homologous plant proteins confirmed the presence of seven metal-binding residues in five conserved motifs [***D***XG/G***D***XX***Y***/G***N***H(D/E)/VXX***H***/G***H***X***H***; bold italic letters represent invariant residues], which is characteristic of plant PAPs (Supplementary Fig. S1).

**Fig. 3.**
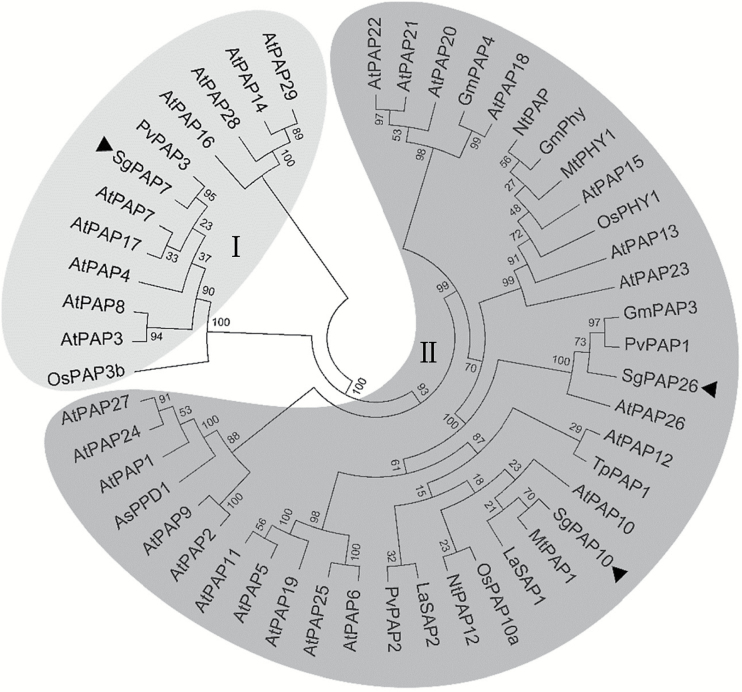
Phylogenetic analysis of plant PAP proteins. The phylogenetic tree was constructed using the neighbour-joining method with 1000 bootstrap replicates in the MEGA 5 program. The first two letters of each PAP protein label represent the abbreviated species name. As: *Astragalus sinicus*; At: *Arabidopsis thaliana*; Gm: *Glycine max*; La: *Lupinus albus*; Mt: *Medicago truncatula*; Nt: *Nicotiana tabacum*; Os: *Oryza sativa*; Pv: *Phaseolus vulgaris*; Sg: *Stylosanthes guianensis*; Tp: *Tagetes patula*. Roman numerals I and II designate the two groups of PAP proteins. Bootstrap values are indicated for major branches as percentages. The three SgPAPs are indicated by black triangles.

A neighbour-joining phylogenetic tree was constructed for plant PAPs, including SgPAP7, SgPAP10, and SgPAP26 from stylo; all PAP members in Arabidopsis, and other PAP members in rice, bean, tobacco, *Medicago truncatula*, soybean, astragalus (*Astragalus sinicus*), white lupin (*Lupinus albus*), and dwarf marigold (*Tagetes patula*). In the resulting phylogenetic tree, plant PAPs were widely classified into two groups, group I with low molecular mass PAPs and group II with high molecular mass PAPs (Fig. 3). Among stylo PAPs, SgPAP7 belonged to group I, with the highest similarity to PvPAP3, whereas SgPAP10 and SgPAP26 were classified into group II, including AtPAP10 and AtPAP26 (Fig. 3).

### Subcellular localization of SgPAPs

**Fig. 4.**
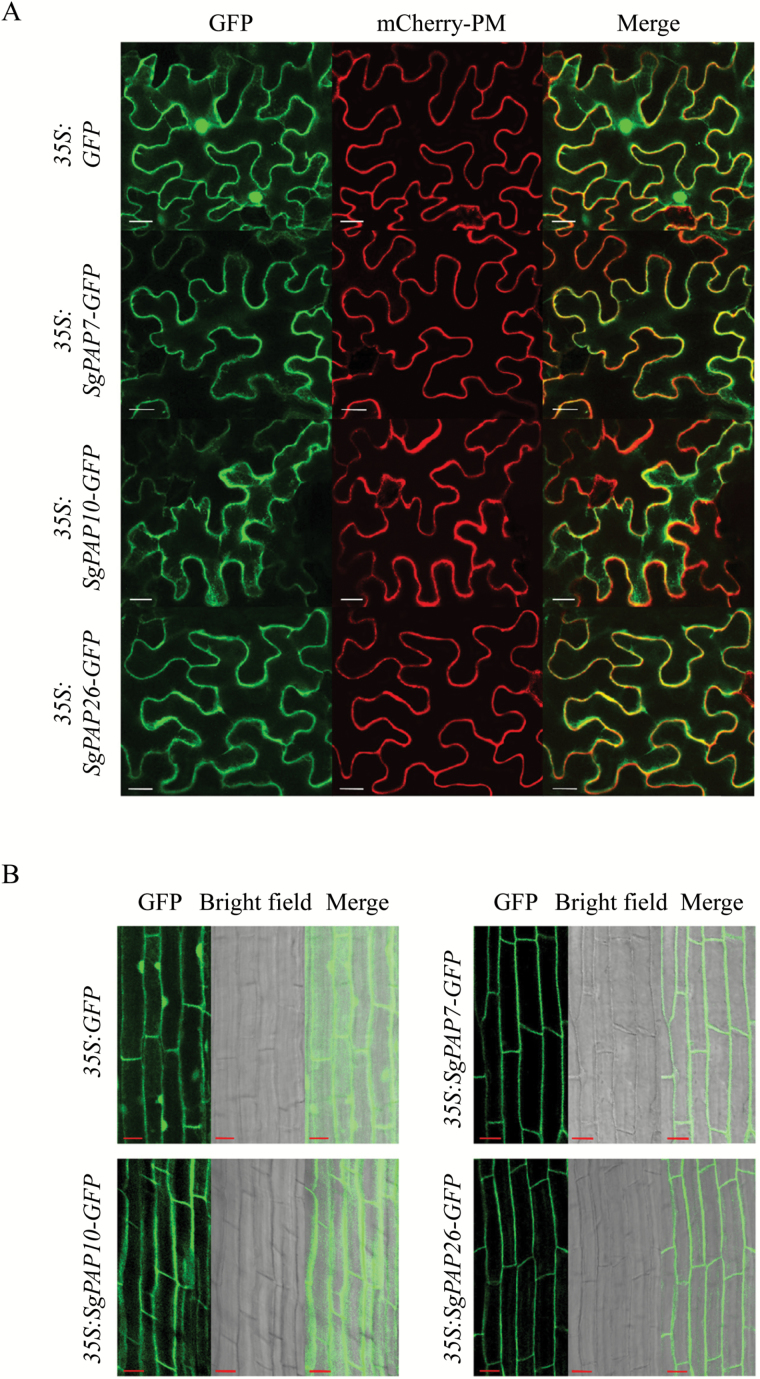
Subcellular localization of SgPAPs. (**A**) Transient expression in tobacco leaf epidemic cells, with bars = 20 µm. (**B**) GFP fluorescence analysis of transgenic bean hairy roots, with bars =10 µm. The images are labelled to show the empty vector control and the *35S:SgPAP7-GFP*, *35S:SgPAP10-GFP*, and *35S:SgPAP26-GFP* constructs. GFP fluorescence and mCherry fluorescence of the plasma membrane marker (mCherry-PM) were observed using confocal microscopy.

To investigate the subcellular localization of SgPAP7, SgPAP10, and SgPAP26, these encoding regions were separately fused to *GFP* at the C-terminus, and the constructs were transiently expressed in tobacco leaf epidermal cells and Arabidopsis mesophyll protoplasts. Localization was determined by the detection of GFP signals in confocal laser scanning microscopy analysis (Fig. 4A; Supplementary Fig. S2). The GFP signal of the empty vector control was detected in the plasma membrane, cytoplasm, and nucleus (Fig. 4A; Supplementary Fig. S2), whereas GFP signals from fusion with SgPAP7, SgPAP10, and SgPAP26 were mainly detected in the plasma membrane (Fig. 4A; Supplementary Fig. S2). GFP fluorescence from all the SgPAP fusions was also found in the cytoplasm (Fig. 4A; Supplementary Fig. S2).

To further confirm the subcellular localization of SgPAP7, SgPAP10, and SgPAP26, the coding regions fused with the *GFP* were also overexpressed in transgenic bean hairy roots. As shown in Fig. 4B, the GFP signal was mainly localized to the cell periphery in transgenic bean hairy roots overexpressing *SgPAP7-GFP*, *SgPAP10-GFP*, or *SgPAP26-GFP* (Fig. 4B), while fluorescence was also observed in the cytoplasm (Fig. 4B). Furthermore, soluble protein and membrane protein were separately extracted from transgenic bean hairy roots overexpressing *SgPAP-GFP*, and analysed by western blot to verify their subcellular localization. Results showed that soluble proteins and membrane proteins were successfully separated because phosphoenolpyruvate carboxylase (PEPC), a cytoplasmic protein marker, and ATPase, a plasma membrane protein marker, were only detected in soluble protein and membrane protein fractions, respectively (see Supplementary Fig. S3A, B). However, SgPAP7-GFP, SgPAP10-GFP, and SgPAP26-GFP were detected in both soluble protein and membrane protein fractions (Supplementary Fig. S3C), suggesting that SgPAP7, SgPAP10, and SgPAP26 could be co-localized in the plasma membrane and cytoplasm.

### Transcriptional responses of SgPAPs to Pi starvation

**Fig. 5.**
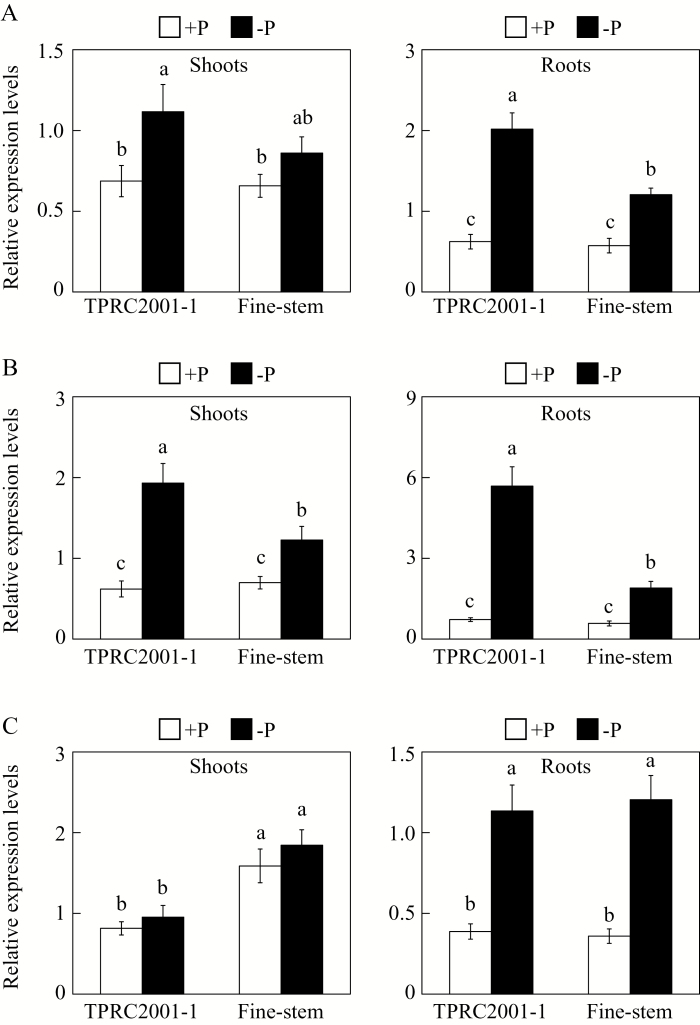
Transcriptional responses of *SgPAPs* to Pi deprivation in two stylo genotypes. (**A**) Expression levels of *SgPAP7.* (**B**) Expression levels of *SgPAP10*. (**C**) Expression levels of *SgPAP26*. After seedlings were grown on +P (1.2mM KH_2_PO_4_) or −P (0 µM KH_2_PO_4_) MS medium for 7 d, total RNA isolated from shoots and roots was used for qRT-PCR analysis. Relative expression levels of the *SgPAPs* are shown relative to the housekeeping gene *SgEF-1a*, using arbitrary units. Each bar represents the mean with standard error of four replicates. Significant differences (*P* < 0.05) are indicated by different letters.

To examine whether expression levels of *SgPAP7*, *SgPAP10*, and *SgPAP26* were responsive to Pi starvation in stylo, their expression patterns were investigated in both shoots and roots of TPRC2001-1 and Fine-stem grown in +P or −P treatments. The results showed that, except for *SgPAP7* expression in Fine-stem shoots, transcription levels of *SgPAP7* and *SgPAP10* were significantly increased by Pi starvation in both shoots and roots of these two stylo genotypes (Fig. 5A, B). In contrast, transcription levels of *SgPAP26* were increased by Pi starvation in roots of both genotypes, but exhibited no response to Pi starvation in shoots of either genotype (Fig. 5C). Furthermore, expression levels of *SgPAP7* and *SgPAP10* were higher in TPRC2001-1 than in Fine-stem at low P levels, especially in roots, where expression of *SgPAP7* and *SgPAP10* in TPRC2001-1 was 67% and 200% higher than in Fine-stem, respectively (Fig. 5A, B).

### SgPAP overexpression results in increased root APase activities

**Fig. 6.**
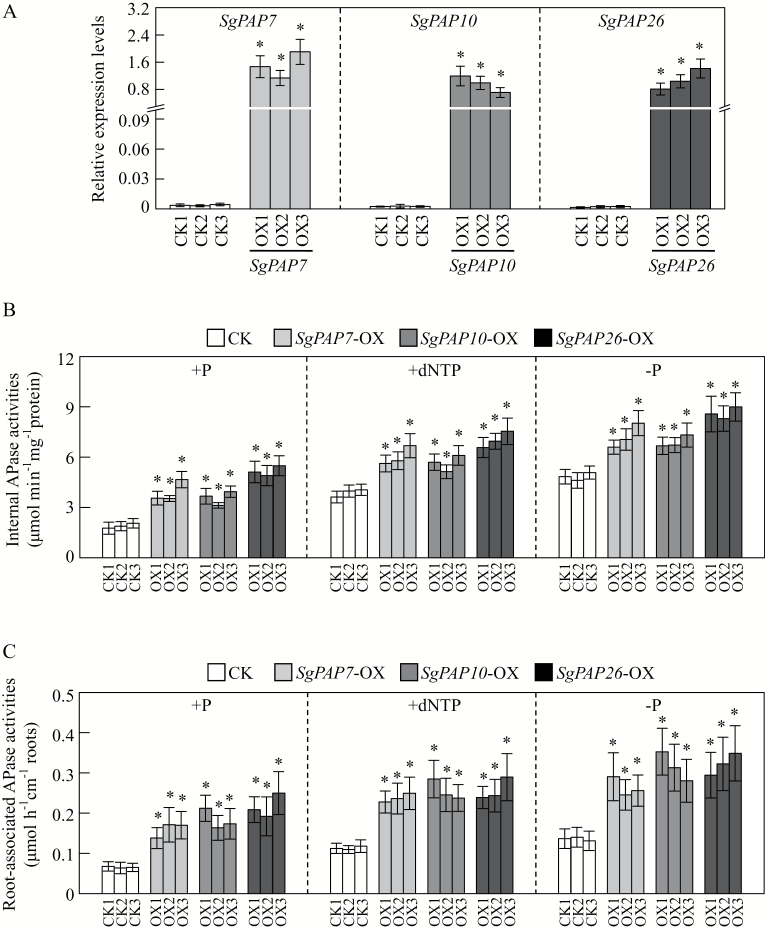
Transcripts and APase activities in transgenic bean hairy roots overexpressing *SgPAPs*. (**A**) Relative expression levels of transcripts of *SgPAPs* in bean hairy roots grown under phosphate application conditions as determined by qRT-PCR analysis. (**B**) Internal APase activities. (**C**) Root-associated APase activities. CK1, CK2, and CK3 represent three transgenic hairy root lines transformed with the empty vector. OX1, OX2, and OX3 indicate transgenic hairy root lines overexpressing *SgPAP7*, *SgPAP10*, or *SgPAP26*, respectively. Transgenic bean hairy roots were grown on MS medium supplied with 1.2mM KH_2_PO_4_ (+P), 0.4mM dNTP (+dNTP), or 0 µM KH_2_PO_4_ (−P) as the sole P source for 14 d. Each bar represents the mean of four biological replicates with standard error. Asterisks indicate significant differences between *SgPAP* overexpression lines and CK lines (*P* < 0.05).

**Fig. 7.**
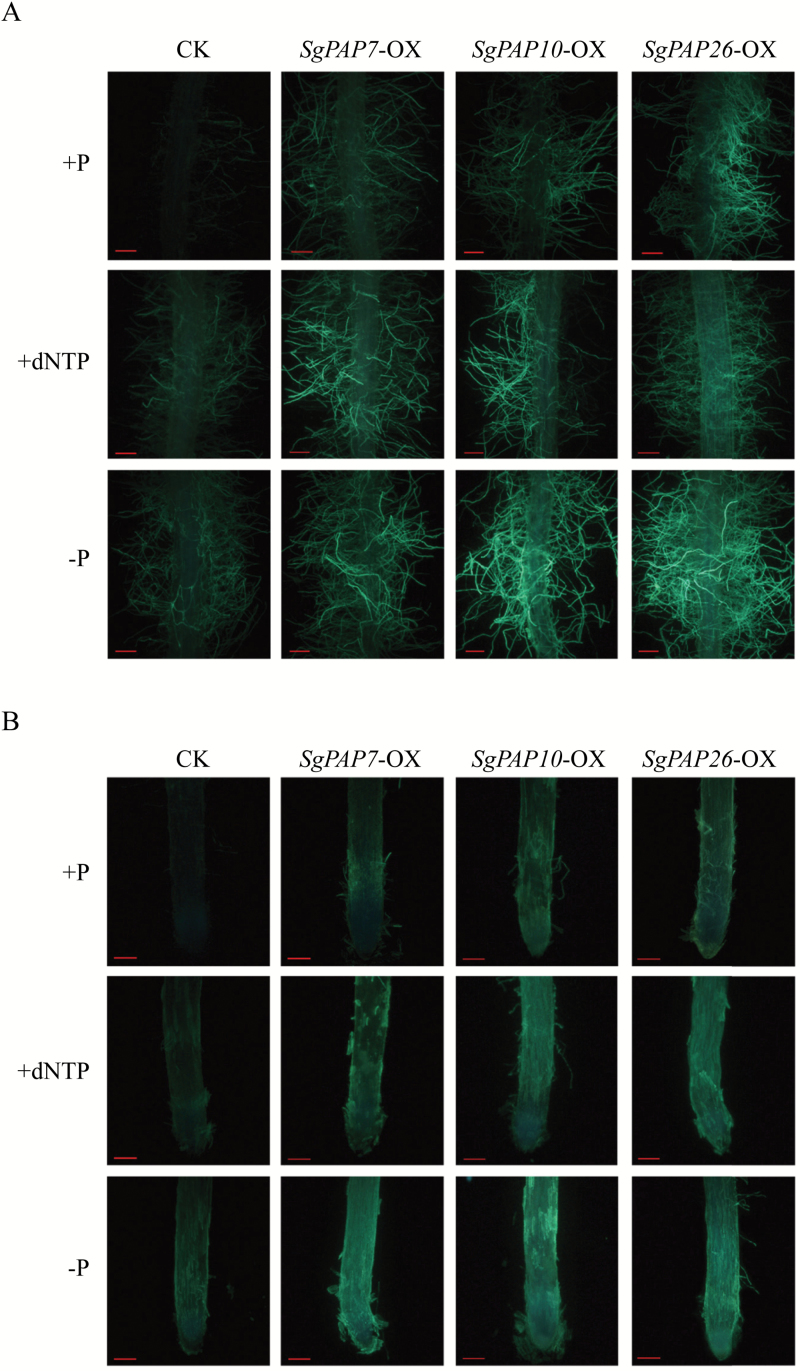
Histochemical staining of root-associated APase activities in bean hairy roots using ELF-97 phosphate as the substrate. (**A**) Detection of root-associated APase activities in the root hair zone. (**B**) Detection of root-associated APase activities in the root tip. Hairy roots were incubated in ELF-97 phosphate solution for 30min. Fluorescence was examined using fluorescence microscopy. Bars = 200 µm. Transgenic hairy roots cultivated on MS medium containing 1.2mM KH_2_PO_4_ (+P), 0.4mM dNTP (+dNTP), or 0 µM KH_2_PO_4_ (−P) for 14 d, before being used for staining of root-associated APase activities. CK indicates transgenic bean hairy roots transformed with the empty vector. *SgPAP7*-OX, *SgPAP10*-OX, and *SgPAP26*-OX indicate transgenic hairy roots overexpressing *SgPAP7*, *SgPAP10*, and *SgPAP26*, respectively.

To elucidate the functions of *SgPAP7*, *SgPAP10*, and *SgPAP26*, the three *SgPAPs* were separately overexpressed (indicated by -OX) in transgenic bean hairy roots, with overexpression confirmed by qRT-PCR analysis (Fig. 6A). Overexpression of *SgPAP7*, *SgPAP10*, and *SgPAP26* led to significant increases of internal APase activities in the transgenic bean hairy roots compared to those in the control lines with application of phosphate (+P) or dNTP (+dNTP) or without phosphate application (−P) (Fig. 6B). Furthermore, root-associated APase activities were also increased relative to those in controls by more than 75% in *SgPAP7*-OX lines, 100% in *SgPAP10*-OX lines, and 102% in *SgPAP26*-OX lines (Fig. 6C). To further validate root-associated APase activities, ELF-97 phosphate and BCIP were used as substrates to visualize root-associated APase activities *in vivo* (Fig. 7; Supplementary Fig. S4). When using ELF-97 phosphate as the substrate, in control bean hairy root lines grown in +P treatment, green fluorescence was weak and negligible in the root tip and in root hair (Fig. 7). In contrast, green fluorescence was more intense in both the root tip and root hairs of bean hairy roots overexpressing *SgPAP7*, *SgPAP10*, or *SgPAP26* with phosphate application (Fig. 7). More intense green fluorescence was also observed in *SgPAPs*-OX lines than in control lines under −P and +dNTP conditions (Fig. 7). Similar results were observed when analysing root-associated APase activities in bean hairy roots using BCIP as the substrate (see Supplementary Fig. S4). A more intense blue colour was observed along root surfaces of *SgPAP7*-OX, *SgPAP10*-OX, and *SgPAP26*-OX lines when compared to staining in control lines, particularly for *SgPAP10*-OX and *SgPAP26*-OX lines (Supplementary Fig. S4), which strongly suggests that overexpression of *SgPAP7*, *SgPAP10*, or *SgPAP26* can contribute to significant increases of root-associated APase activities in bean hairy roots.

### Overexpressing SgPAPs enhances extracellular dNTP utilization

**Fig. 8.**
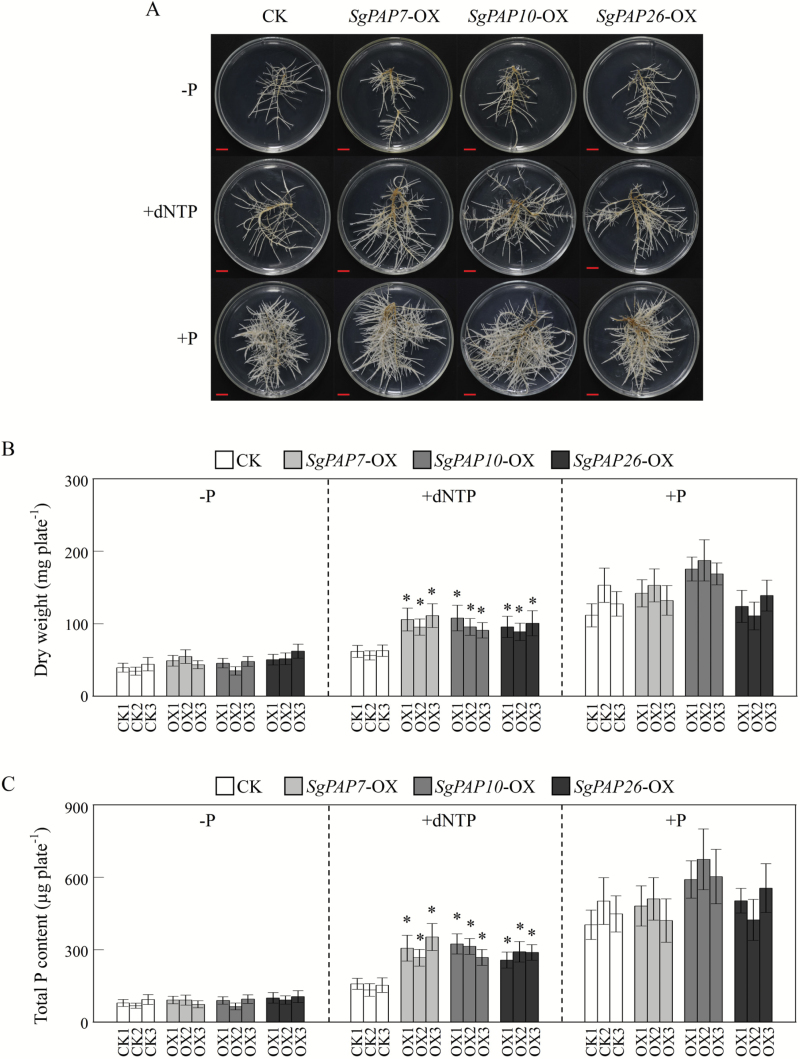
Growth and total P content of transgenic bean hairy roots supplied with different P sources. (**A**) Photograph of transgenic bean hairy roots supplied with different P sources. Bars = 1cm. (**B**) Dry weight in transgenic bean hairy roots. (**C**) Total P content in transgenic bean hairy roots. Transgenic bean hairy roots were grown on MS medium supplied with 0 µM KH_2_PO_4_ (−P), 0.4mM dNTP (+dNTP), or 1.2mM KH_2_PO_4_ (+P) as the sole P source for 14 d. Dry weight and total P content were separately measured. CK1, CK2, and CK3 represent three transgenic hairy root lines transformed with the empty vector. OX1, OX2, and OX3 indicate lines overexpressing *SgPAP7*, *SgPAP10*, and *SgPAP26*, respectively. Each bar is the mean of four biological replicates with standard error. Asterisks indicate significant differences between *SgPAP* overexpression lines and CK lines (*P* < 0.05).

To evaluate functions of *SgPAP7*, *SgPAP10*, and *SgPAP26* involved in extracellular dNTP utilization, bean hairy roots were grown on MS medium with or without dNTP application. Without dNTP application, no significant difference was observed in appearance or dry weight among transgenic lines of bean hairy roots grown in either −P or +P treatments (Fig. 8A, B). However, with dNTP application, dry weight in *SgPAP7*, *SgPAP10* and *SgPAP26* overexpression bean hairy roots was more than 39% higher than in control lines (Fig. 8A, B). Consistent with changes of dry weight, when dNTP was used as the sole external P source in the growth medium, total P content increased by more than 68% in *SgPAP7*-OX lines, 69% in *SgPAP10*-OX lines, and 62% in *SgPAP26*-OX lines, compared with total P content in control lines (Fig. 8C). Yet, consistent with dry weight results, total P content among lines of bean hairy roots was similar in either the −P or +P treatments (Fig. 8C). This result suggests that *SgPAP7*, *SgPAP10*, and *SgPAP26* can participate in extracellular dNTP utilization in stylo.

## Discussion

To cope with P deficiency, plants have developed a set of morphological and physiological strategies that are coordinately regulated by complex P signalling networks (Chiou and Lin, 2011; Liang *et al.*, 2014; Sun *et al.*, 2016). Although most adaptive strategies are well conserved in plants, significant variations have been observed across or within plant species for several adaptive responses, especially for alteration of root morphology and architecture (López-Arredondo *et al.*, 2014). For example, it is generally observed that low P inhibits primary root growth in Arabidopsis, but not in other plant species, such as barley (*Hordeum vulgare*) and rice (Zhou *et al.*, 2008; Péret *et al.*, 2014). However, unlike various changes in root morphology and architecture, it seems that Pi-starvation-caused increases in APase activities are well conserved in a wide range of plant species, such as Arabidopsis, bean, soybean, wheat (*Triticum aestivum*), barley, and rice. It is therefore thought to be an important adaptive strategy in plants responsive to P deficiency (Tadano and Sakai, 1991; Asmar *et al.*, 1995; Yan *et al.*, 2001; George *et al.*, 2008; Tran *et al.*, 2010a). Consistent with this hypothesis, both root internal and root-associated APase activities were significantly increased by Pi starvation in two stylo genotypes (Fig. 2), suggesting that stylo, like other plant species, can adapt to P deficiency via increased APase activities.

It has been documented through forward genetic, biochemical, and protein MS analyses that increased APase activities are mainly due to increased transcripts or post-transcriptional modification of *PAP* members in plants under low P conditions. In Arabidopsis, knockout of *AtPAP12* and *AtPAP26* led to nearly 50% and 30% decreases, respectively, in intracellular APase activities under P-deficient conditions (Wang *et al.*, 2014). Furthermore, *atpap12/atpap26* double mutants displayed a 60% reduction in root-secreted APase activities under low P conditions (Robinson *et al.*, 2012; Wang *et al.*, 2014), suggesting that enzymatic activities of PAPs contribute to APase activities in plants.

Consistent with this conclusion, biochemical and MS analyses of purified APase isoforms also revealed that increased APase activities are mainly caused by increased enzymatic activities of plant PAPs, such as AtPAP12 and AtPAP26 in Arabidopsis, LeSAP1 and LeSAP2 in tomato (*Lycopersicon esculentum*), PvPAP3 in bean, LaSAP in white lupin, and NtPAP in tobacco (Miller *et al.*, 2001; Bozzo *et al.*, 2004; Lung *et al.*, 2008; Liang *et al.*, 2010; Tran *et al.*, 2010b). For example, a Pi-starvation-induced APase isoform was purified from bean roots, and identified as PvPAP3 by sequence analysis (Liang *et al.*, 2010). It has been suggested that increased *PvPAP3* transcripts lead to PvPAP3 accumulation, and thus increase APase activities in both bean roots and leaves under low P conditions (Liang *et al.*, 2010). In this study, *SgPAP7*, *SgPAP10*, and *SgPAP26* were cloned from stylo, and found to be highly homologous with *PvPAP3* in bean, and *AtPAP10* and *AtPAP26* in Arabidopsis, respectively (Fig. 3). It was observed that root internal and root-associated APase activities increased with Pi starvation, which was accompanied by increased transcripts of *SgPAP7*, *SgPAP10*, and *SgPAP26* (Figs 2 and 5). Furthermore, overexpression of *SgPAP7*, *SgPAP10*, or *SgPAP26* in bean hairy roots led to increases in internal and root-associated APase activities of more than 27% and 75%, respectively (Fig. 6B, C). These results strongly suggest that increased transcripts of *SgPAP7*, *SgPAP10*, or *SgPAP26* can contribute to increased APase activities in stylo under low P conditions.

Functions of plant PAPs are partially determined by their subcellular localization. It has been well documented that plant PAPs can localize in many subcellular regions, including the cell wall, vacuole, nucleus, plastids, mitochondria, apoplast, and secretome, which implies complex and diverse functions for plant PAPs (Li *et al.*, 2008; Kaida *et al.*, 2009; Hurley *et al.*, 2010; Liang *et al.*, 2010, 2012; Sun *et al.*, 2012; Del Vecchio *et al.*, 2014). However, plasma membranes have been found to harbour only a few plant PAPs, such as PvPAP1 and PvPAP3 in bean, and AsPPD1 in *Astragalus sinicus* (Liang *et al.*, 2010, 2012; Wang *et al.*, 2015).

Through bioinformatic analysis, a transmembrane helix was found in the topology of SgPAP7, SgPAP10, and SgPAP26 (see Supplementary Fig. S5), suggesting that these three SgPAPs might localize on the plasma membrane. That these three SgPAPs were localized on the plasma membrane was consistently observed through transient expression analysis of *SgPAP*-*GFP* in tobacco leaf epidermal cells and Arabidopsis mesophyll protoplasts, as well as overexpression analysis of *SgPAP-GFP* in transgenic bean hairy roots (Fig. 4A, B; Supplementary Fig. S2). Like SgPAP7, its ortholog PvPAP3 has been found to be localized on the plasma membrane through transient expression of *PvPAP3*-*GFP* in onion epidermal cells (Liang *et al.*, 2010), which suggests, along with sequence similarity, that PvPAP3 and SgPAP7 perform similar functions in plants. Although SgPAP26 exhibited similar subcellular localization to PvPAP1, its ortholog in bean (Liang *et al.*, 2012), variation in subcellular localization from other orthologs has been found, including dual-targeting to both the cell wall and vacuole for AtPAP26 in Arabidopsis, and to the mitochondria for GmPAP3 in soybean (Li *et al.*, 2008; Hurley *et al.*, 2010; Robinson *et al.*, 2012). This suggests that the diverse functions of PAPs in plants are attributable to the variation in their subcellular localization.

It has been demonstrated that soil microorganisms play a key role in soil–plant P cycling, with activities including immobilization of Pi from the soil solution, mineralization of soil organic P, and incorporation of Pi into microbial biomass (Richardson *et al.*, 2011). It is estimated that microbial P levels can reach 30–40% of the total soil P, which mainly consists of nucleic acids (Achat *et al.*, 2010). Therefore, plants might be capable of utilizing extracellular nucleic acids (e.g. DNA and dNTP). Recently, utilization of nucleic acid P and other phosphomonoesters has been reported for a variety of plant PAPs, such as PvPAP3 in bean, OsPAP10a in rice, and AtPAP12 and AtPAP26 in Arabidopsis (Liang *et al.*, 2010; Robinson *et al.*, 2012; Tian *et al.*, 2012; Tian and Liao, 2015). In Arabidopsis, the biomass of *atpap12/atpap26* mutant plants was significantly lower than that of wild-type plants when DNA was supplied as the sole P source in growth medium, suggesting that both AtPAP12 and AtPAP26 might be involved in the utilization of extracellular DNA (Robinson *et al.*, 2012). PvPAP1 and PvPAP3 in bean were also demonstrated to augment extracellular dNTP utilization (Liang *et al.*, 2012). In the current study, when dNTP was supplied as the sole extracellular P source, significant increases of both total P content and soluble Pi concentration were observed in two stylo genotypes (Fig. 1), suggesting that stylo is able to utilize exogenous dNTP. To elucidate molecular mechanisms underlying stylo utilization of extracellular dNTP, homologues of *PvPAP3*, *AtPAP10*, and *AtPAP26* in stylo were cloned and named *SgPAP7*, *SgPAP10*, and *SgPAP26*. The significant increases in root-associated APase activities as well as the higher dry weight and total P content in transgenic bean hairy roots overexpressing *SgPAP7*, *SgPAP10*, or *SgPAP26* when dNTP was supplied as the sole extracellular P source (Figs 6, 7, and 8) strongly suggest that these genes participate in extracellular dNTP utilization in stylo. Furthermore, low-P-enhanced higher expression levels of *SgPAP7* and *SgPAP10* in TPRC2001-1 than in Fine-stem (Fig. 5) might facilitate the utilization of more extracellular dNTP for TPRC2001-1 than for Fine-stem (Fig. 1).

Taken together, the results herein strongly suggest that Pi starvation can increase root-associated APase activities in stylo, which might be caused by enhanced expression levels of *SgPAP7*, *SgPAP10*, and *SgPAP26* in roots of both stylo genotypes. Furthermore, higher expression levels of *SgPAP7* and *SgPAP10* in TPRC2001-1 roots contribute to its higher root-associated APase activities, and thus facilitate greater utilization of extracellular dNTP by TPRC2001-1 than by Fine-stem.

## Supplementary material

Table S1. A list of primers used in the study.

Figure S1. Multiple sequence alignment of three SgPAPs and orthologous plant PAPs.

Figure S2. Subcellular localization of SgPAPs in Arabidopsis mesophyll protoplasts.

Figure S3. Western-blot analysis of soluble protein and membrane protein extracts from transgenic bean hairy roots.

Figure S4. Histochemical staining of root-associated APase activities in bean hairy roots using BCIP as the substrate.

Figure S5. Prediction of SgPAP transmembrane topology.

Supplementary Data
