# Supplementary material for: Characterization of purple acid phosphatases involved in extracellular dNTP utilization in Stylosanthes
Source: J Exp Bot. 2016 May 18;67(14):4141–54. doi: 10.1093/jxb/erw190 (PMC5301924; doi:10.1093/jxb/erw190)
Supplement: Supplementary Data [file supp_67_14_4141__index.html]

Characterization of purple acid phosphatases involved in extracellular dNTP utilization in Stylosanthes — Characterization of purple acid phosphatases involved in extracellular dNTP utilization in Stylosanthes — Supplementary Data 

# Characterization of purple acid phosphatases involved in extracellular dNTP utilization in *Stylosanthes*

## Supplementary Data

Data files

- supplementary\_figures\_S1\_S5\_table\_S1.pdf - Supplementary Data
