# Supplementary material for: Characterization of purple acid phosphatases involved in extracellular dNTP utilization in Stylosanthes
Source: J Exp Bot. 2016 May 18;67(14):4141–54. doi: 10.1093/jxb/erw190 (PMC5301924; doi:10.1093/jxb/erw190)
Supplement: Supplementary Data [file supp_erw190_supplementary_figures_S1_S5_table_S1.pdf]

Fig. S1

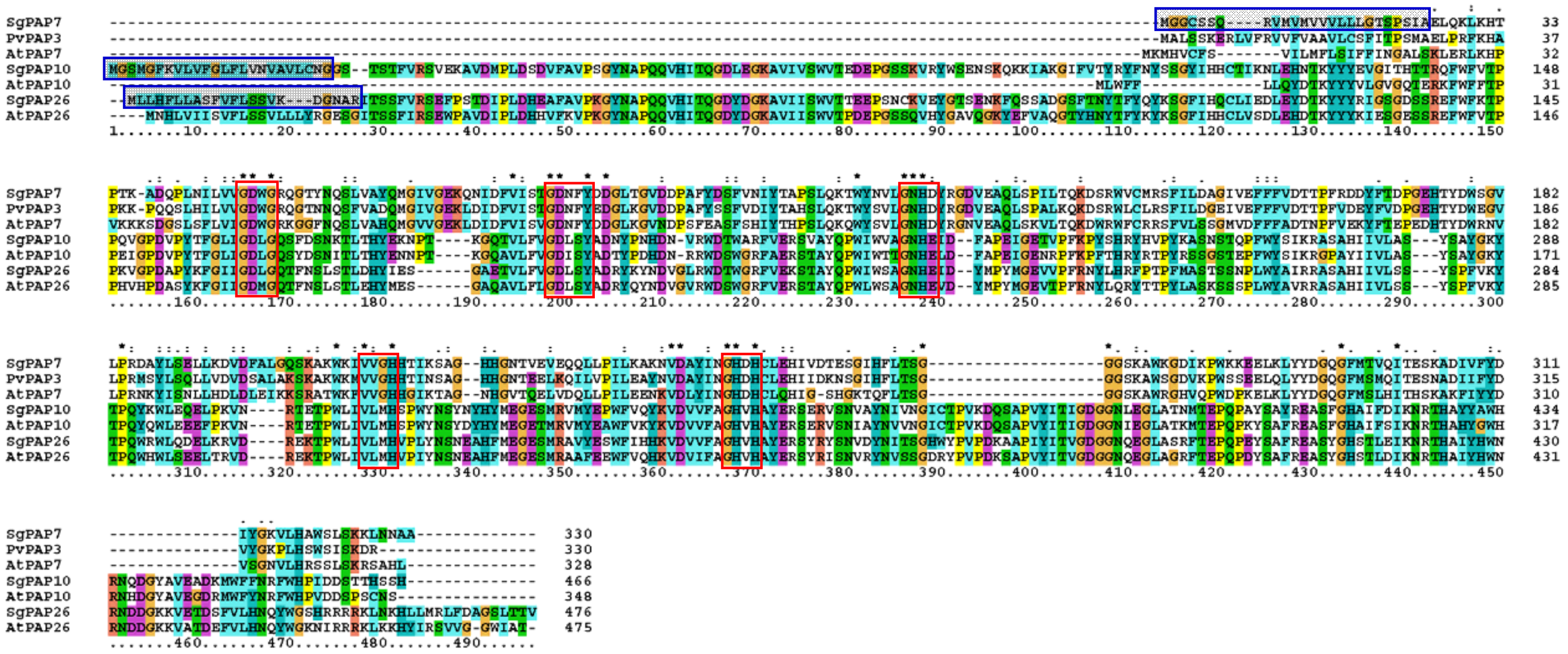

Fig. S1 Multiple sequence alignment of three SgPAPs and orthologous plant PAPs. Red boxes indicate positions of conserved sequence motifs of PAP. Putative signal peptide cleavage sites for SgPAP7, SgPAP10 and SgPAP26 are indicated by blue boxes.

Fig. S2

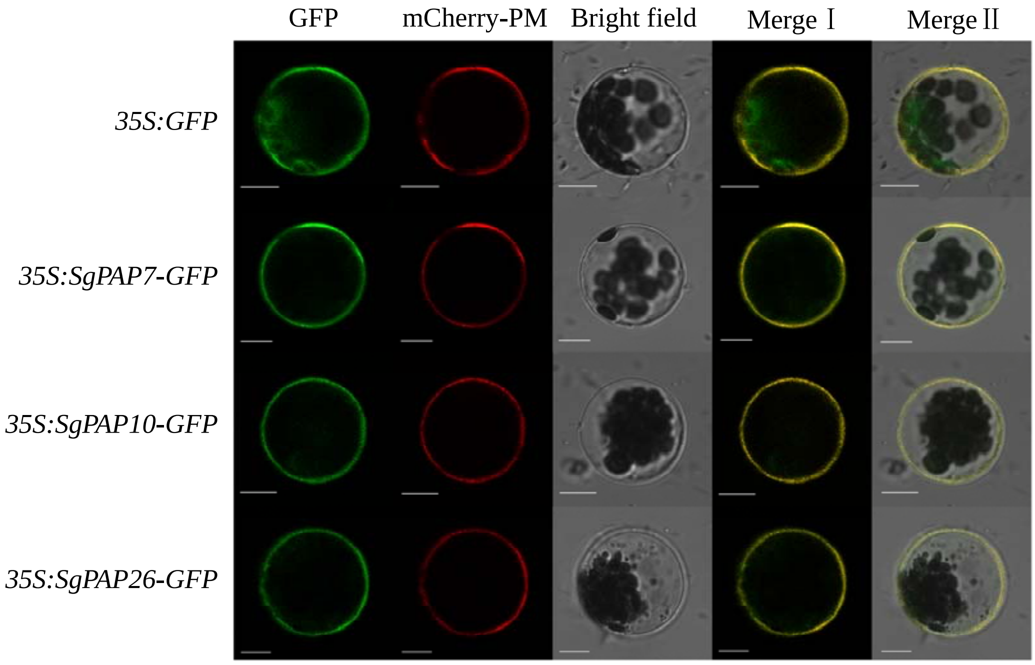

Fig. S2 Subcellular localization of SgPAPs in Arabidopsis mesophyll protoplasts. Protoplasts were transiently co-transfected with the plasma membrane marker (mCherry-PM), along with the empty vector control or SgPAP-GFP. GFP fluorescence is shown in green and mCherry fluorescence in red. The Merge Ⅰ images show a merged view of both fluorescence channels. Merge Ⅱ images include the fluorescent signal panels and the bright field channels. Bars =10 μm.

Fig. S3

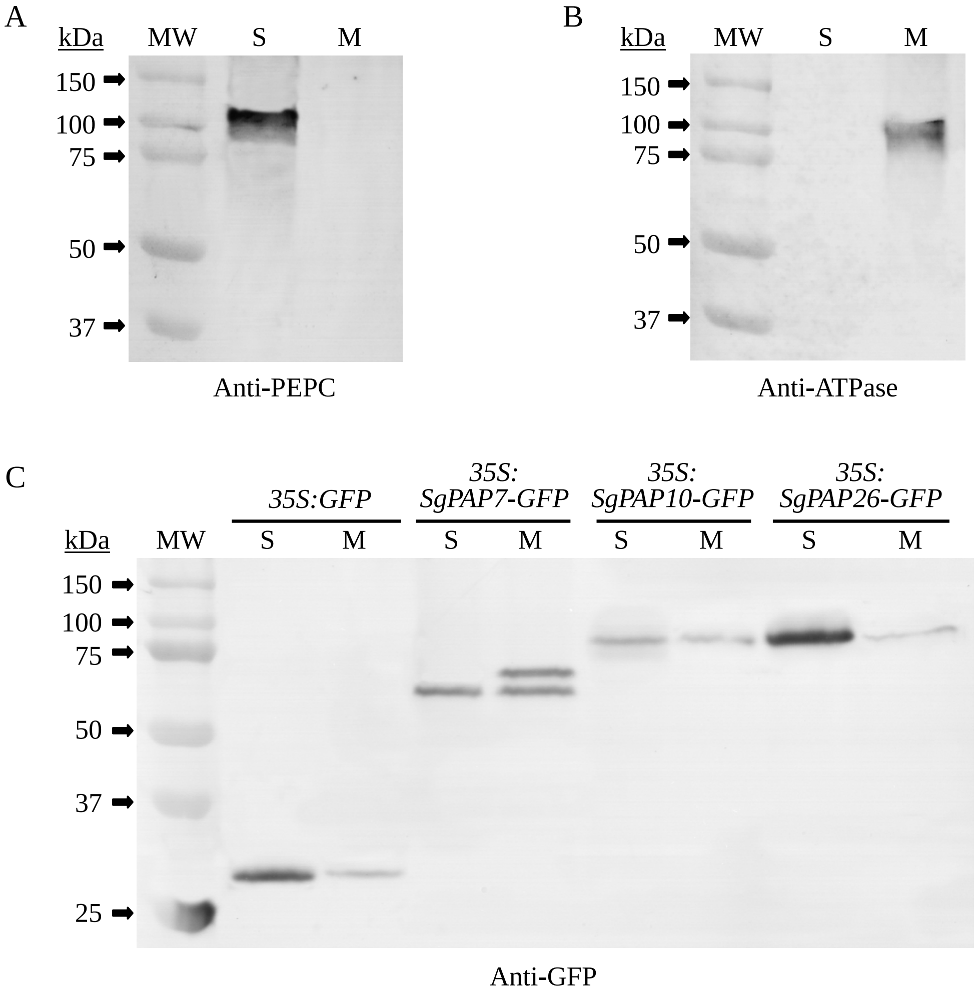

Fig. S3 Western-blot analysis of soluble protein and membrane protein extracts from transgenic bean hairy roots. Soluble proteins and membrane proteins extracted from control lines were subjected to immunoblot analysis using anti-PEPC antibody (A) and anti-ATPase antibody (B). Immunoblotting was also performed for soluble protein and membrane protein extracts from control lines or *SgPAP-GFP* overexpression lines using anti-GFP antibody (C). Totally, 25 μg soluble proteins and 10 μg membrane proteins were separately loaded for each lanes. MW indicates molecular mass standards. S and M represent soluble proteins and membrane proteins, respectively.

Fig. S4

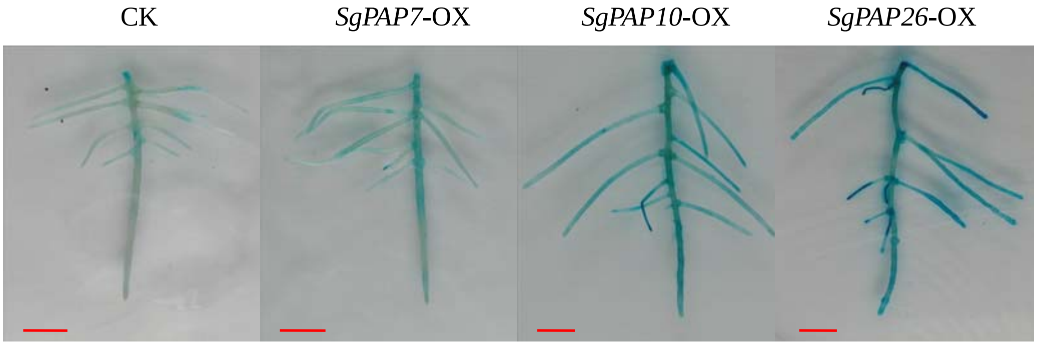

Fig. S4 Histochemical staining of root-associated APase activities in bean hairy roots using BCIP as the substrate. Hairy roots were evenly overlaid with BCIP agar solution for 2 h. Bars =0.5 cm. Transgenic hairy roots cultivated on +P (1.2 mM KH_2PO_4) MS medium were used for staining of root-associated APase activities. CK indicates transgenic bean hairy roots transformed with the empty vector. *SgPAP7*-OX, *SgPAP10*-OX and *SgPAP26*-OX indicate transgenic hairy roots overexpressing *SgPAP7*, *SgPAP10* and *SgPAP26*, respectively.

Fig. S5

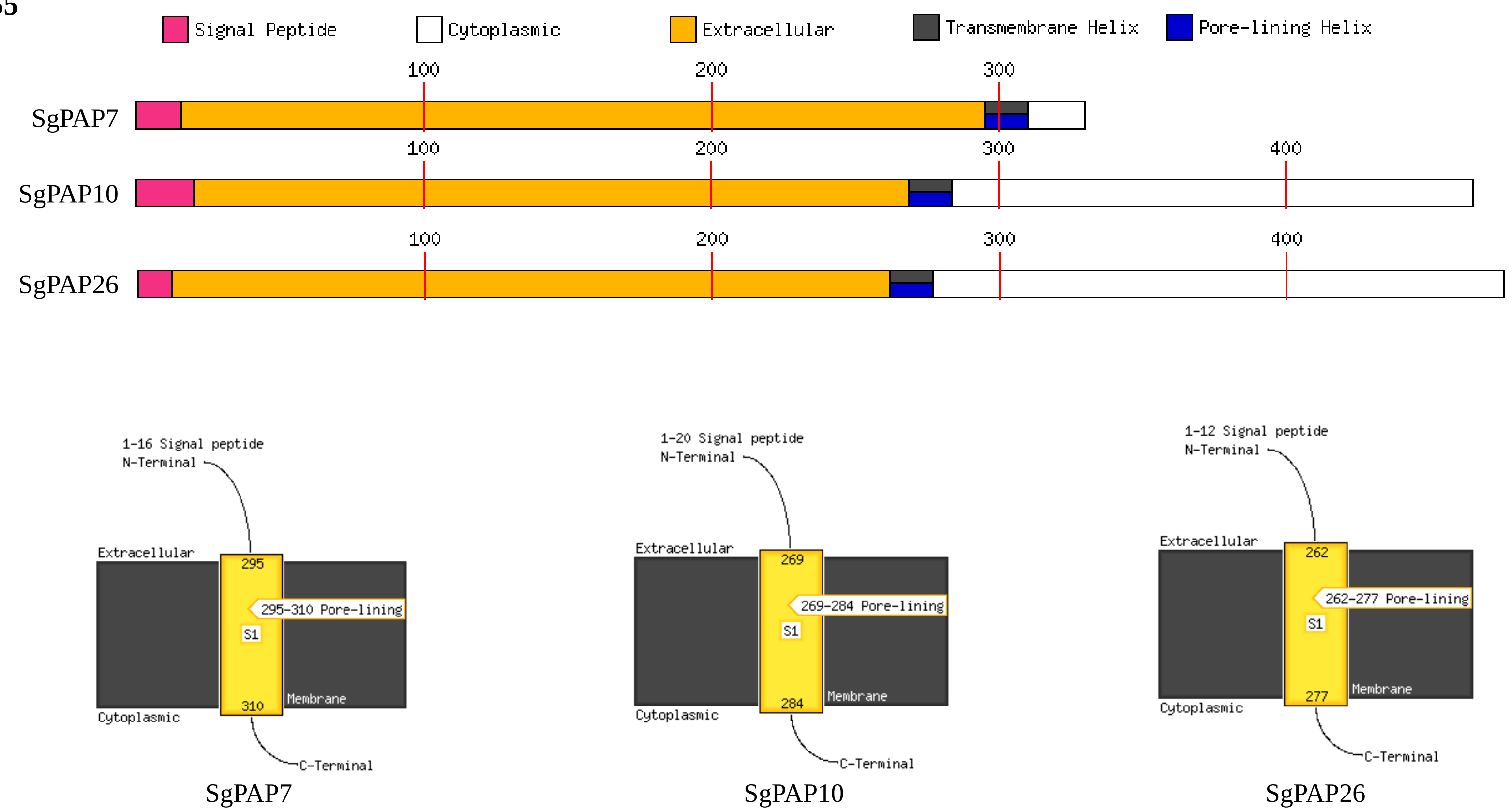

Fig. S5 Prediction of SgPAP trans-membrane topology. Trans-membrane helices of SgPAPs were predicted by MEMSAT-SVM (http://bioinf.cs.ucl.ac.uk/web_servers/).

Table S1 A list of primers used in the study

Primer name	Sequence (5’- 3’)
SgPAP7-EST-F	GTTGATGATCCAGCATTCTAC
SgPAP7-EST-R	GGTCATGTCCATTTATGTATGC
SgPAP10-EST-F	TGGTCTCATTGGGGATCTTG
SgPAP10-EST-R	GCTGTGGTTCTGTCATGTTGGT
SgPAP26-EST-F	CAAGGTGACTATGATGGAAAAGC
SgPAP26-EST-R	CGCATGCTTTCACCCTCCAT
RACE-UPM	CTAATACGACTCACTATAGGGCAAGCAGTGGTATCAACGCAGAGT
RACE-NUP	AAGCAGTGGTATCAACGCAGAGT
SgPAP7-5’-RACE-R	GGTATGCTCTCCCGGGTCCGTAAAGT
SgPAP7-3’-RACE-F	CTTGATGCAGGCATTGTGGAATTCTTC
SgPAP10-5’-RACE-R	CCAAGGCGAATGCATGAGCACAATC
SgPAP10-3’-RACE-F	GGATTTGGGTTGCAGGGAACCATG
SgPAP26-5’-RACE-R	CACGTATCCCACCGCAGACCAACATC
SgPAP26-3’-RACE-F	CTCCTGGGTGACAACAGAAGAACCAAG
SgPAP7-RT-F	CAGCAACTCCTTCCCATCCTAAAG
SgPAP7-RT-R	ATCCACCTCCGCTTGTTAGAAAGT
SgPAP10-RT -F	GCGAGAACAGCAAGCAAAAGAAG
SgPAP10-RT -R	GTGTGTGTGATTCCGACTTCATAGT
SgPAP26-RT-F	AGCGAGTTGACAGGGAGAAGAC
SgPAP26-RT-R	ACCCGCAAAGACCACATCAAC
SgPAP7-OX-F	GAGCTCATGGGGACTTCCAATAATCTCT
SgPAP7-OX-R	ACGCGTTCATGCTGCATTGTTAAGCTT
SgPAP10-OX -F	CCCAAGCTTATGGGTTCAATGGGTTTCAAAGT
SgPAP10-OX- R	CGACGCGTTTAATGAGAACTGTGAGTTGTGG
SgPAP26-OX -F	CCCAAGCTTATGTTGCTTCATTTTTTGCTAGCT
SgPAP26-OX -R	CGACGCGTTTAGACGGTGGTAAGACTACCAGC
SgPAP7-GFP -F	GCTCTAGAG ATGGGTGGGTGTTCAAGCC
SgPAP7-GFP -R	GGGGTACCGT TGCTGCATTGTTAAGCTTTTTGG
SgPAP10-GFP -F	GCTCTAGAG ATGGGTTCAATGGGTTTCAAAGT
SgPAP10-GFP -R	GGGGTACCGT ATGAGAACTGTGAGTTGTGGAATC
SgPAP26-GFP -F	GCTCTAGAG ATGTTGCTTCATTTTTTGCTAGCT
SgPAP26-GFP -R	GGGGTACCGT GACGGTGGTAAGACTACCAGCAT
SgEF-1a-F	CACTTCAGGACGTGTACAAGATC
SgEF-1a-R	CTTGGAGAGCTTCATGGTGCA
PvEF-1a-F	TGAACCACCCTGGTCAGATT
PvEF-1a-R	TCCAGCATCACCATTCTTCA
